# Evaluating the Antiviral Activity of Termin-8 and Finio Against a Surrogate ASFV-like Algal Virus

**DOI:** 10.3390/pathogens14070672

**Published:** 2025-07-08

**Authors:** Amanda Palowski, Francisco Domingues, Othmar Lopez, Nicole Holcombe, Gerald Shurson, Declan C. Schroeder

**Affiliations:** 1 Department of Veterinary Population Medicine, College of Veterinary Medicine, University of Minnesota, St. Paul, MN 55108, USA; borch143@umn.edu; 2 Anitox Corporation, Lawrenceville, GA 30043, USA; fdomingues@anitox.com (F.D.); olopez@anitox.com (O.L.); nholcombe@anitox.com (N.H.); 3 Department of Animal Science, College of Food Agricultural and Natural Resource Sciences, University of Minnesota, St. Paul, MN 55108, USA; shurs001@umn.edu

**Keywords:** African swine fever virus, Emiliania huxleyi virus, NCLDVs, viral inactivation, viability PCR

## Abstract

The objective of this study was to evaluate the time-course of incubation for the potential preventative mitigation of megaviruses using Termin-8 (a formaldehyde-based product) and Finio (non-formaldehyde solution) from Anitox. Emiliania huxleyi virus (EhV), an algal surrogate for African swine fever virus (ASFV), was treated with the recommended concentrations of Termin-8 (0.1% to 0.3%) and Finio (0.05% to 0.2%), and both viability qPCR (V-qPCR) and standard PCR (S-qPCR) were used to quantify EhV concentrations at 1 h, 5 h, 24 h and day 7 post-inoculation. Overall, Finio, and to a lesser extent Termin-8, at their highest treatment concentrations, showed the greatest log reduction of 4.5 and 2 log_10_ units, respectively, at 1 h post-inoculation. Although Termin-8 efficacy did not improve with time, due to its fixing of viral particles and rendering them non-infectious, treatment with Finio showed 100% viable viral inactivation (>5 log_10_ reduction units) at the lowest concentration after 7 days of exposure. Our results demonstrate that both Termin-8 and Finio can be used as effective chemical mitigants against megaviruses such as EhV and ASFV and can be used as effective preventive or mitigation strategies to prevent the transmission of ASFV by reducing particle viability in contaminated feed, although additional research is warranted.

## 1. Introduction

The United States livestock and poultry industries are under constant threat of transmission of foreign animal diseases. Arguably, the most concerning foreign animal disease threat to the U.S. is African swine fever (ASF), with the etiological agent being a double-stranded DNA megavirus, African swine fever virus (ASFV) [1,2]. The introduction of ASF to the United States pork population would lead to significant revenue losses in both the pork industry and the corn–soybean industry. African swine fever virus is of particular concern because it is stable in a variety of environments (such as feed ingredients) and at various temperatures [1,2,3,4,5]. Because there is no surveillance or monitoring system to determine viral presence or concentration in the global feed supply chain, there is the possibility that ASFV-contaminated feed ingredients could be imported into the U.S. from ASFV-positive countries. Furthermore, because of the high thermal resilience of ASFV and the detrimental effects of high thermal processing temperatures on the nutritional value (e.g., reduced amino acid digestibility, vitamin potency loss), the use of chemical mitigants to inactivate ASFV in contaminated feed ingredients is emerging as a viable biosecurity strategy in global feed supply chains. However, evaluating the efficacy of chemical mitigants against ASFV is difficult because of the very high biosecurity requirements, its restricted use in a limited number of approved research laboratories, and its high cost. Therefore, a safe and effective, risk-free, in situ, non-animal (RISNA) assay has been developed that utilizes the Emiliania huxleyi virus (EhV) as a surrogate for ASFV, which can be used in studies to evaluate the efficacy of chemical mitigants [3,5].

Both EhV and ASFV are giant double-stranded DNA viruses with similar structural properties [6], functional properties, and inactivation mechanisms, but EhV does not infect humans, animals, or plants, making it a safe surrogate virus for ASFV research [1]. The virions of both EhV and ASFV are similar, with both having a nucleoprotein core structure surrounded by an icosahedral capsid, and they are both enclosed by an external lipid envelope [1,3,7]. Additionally, it has recently been shown that both viruses are thermally stable at temperatures up to 80 °C for 20 min, with similar inactivation kinetics when exposed to high temperatures [3,8]. It is also well-documented that porcine viruses, including ASFV and the algal surrogate EhV, can survive in feed and feed ingredients used in commercial swine diets [1,4,9,10]. Due to all these similarities, utilizing the algal surrogate EhV in the RISNA assay for trials and experiments to evaluate mitigation strategies’ effectiveness is very beneficial and informative because it also involves novel analysis assays, including viability quantitative PCR, flow cytometry, and confocal microscopy, to determine viral presence and structure after treatment [11]. Viability quantitative PCR (V-qPCR) is a useful method to quantify the amount of viable virus in a sample, where viability is defined as an intact virus particle that has the potential to initiate infection.

Anitox is a global feed additive company that develops a wide range of products to control microbial contamination in feed, such as Termin-8 and Finio. Termin-8 is a formaldehyde-based product with established efficacy against *Salmonella* species [12,13]. Finio was launched in 2017 in the EU and is currently undergoing the registration process in the U.S. as a non-formaldehyde solution with a patent-protected formulation comprising novel phytochemical and carboxylic acids. To date, neither Finio nor Termin-8 have been tested for their potential antiviral activity against megaviruses such ASFV and its surrogate algal virus, EhV. Given the limited access and expense of routine chemical mitigation testing with ASFV, we used the EhV surrogate to evaluate the antiviral effects and efficacy of Termin-8 and Finio in this study. Therefore, the objective of this study was to evaluate the time-course of incubation from hours to days to determine the antiviral potential of Termin-8 and Finio as a preventative inactivation treatment of megaviruses such as EhV and ASFV.

## 2. Materials and Methods

### 2.1. Cell Culture and EhV-86 Stock

A culture of Emiliania huxleyi CCMP374 was grown in Alga-Gro^®^ Seawater Medium (Carolina Biological Supplement Company, Burlington, NC, USA) at 15 °C with 18 h/6 h light/dark cycle (ca. 2400 lux) until the concentration of 2 × 10^5^ cells/mL was reached. Isolate EhV-86 was added to E. huxleyi at a multiplicity of infection of 1 and grown in a 15 °C incubator until lysis was observed, which usually occurred after 4 days [7]. The lysate was filtered through a 0.45 µm filter (Nalgene™ Rapid-Flow™ Bottle Top Filters, ThermoFisher Scientific, Waltham, MA, USA) to remove cell debris. This filtration and infection procedure was repeated several times. The filtered lysate was divided into aliquots and kept in the dark at 4 °C until use.

Aliquots of EhV (100 μL containing up to 6 log10 copies/mL) in biological triplicates were treated with manufacturer-recommended concentrations of Termin-8 (0.1%, 0.2%, and 0.3% final concentration) and Finio (0.05%, 0.1% and 0.2% final concentration). The EhV triplicates were exposed to varying concentrations of Termin-8 and Finio for 1 h, 24 h, and 7 days at 20 °C.

### 2.2. Standard qPCR and Viability qPCR Assays

Standard and viability qPCR (S-qPCR and V-qPCR) analyses were conducted for all samples using the methods optimized by Balestreri et al. [3]. For the samples evaluated for virus viability (V) using V-qPCR, 1 μL of PMAxx dye (Biotium Inc., Fremont, CA, USA, 25 µM final concentration) was added and incubated at room temperature in the dark for 10 min on a rocker for optimal mixing per manufacturer guidelines. The mixed V samples were exposed to the light using PMA-Lite device (Biotium Inc, Fremont, CA, USA) for 30 min to cross-link the PMAxx dye to the DNA. The standard (S) samples analyzed using S-qPCR were not treated with PMAxx dye and were used as a control.

A Nucleo Mag^®^ Virus (Takara Bio USA, Inc., San Jose, CA, USA) extraction kit was used to extract DNA from all samples. A quantitative PCR was conducted using Quantinova SYBR Green PCR kit (Qiagen, Carlsbad, CA, USA) with the following conditions: 2 min at 95 °C, followed by 40 cycles of 5 s at 95 °C and 10 s at 60 °C, using the methods of Balestreri et al., 2024 [3]. All PCR assays were conducted using Rotor-gene Q Real-Time PCR (Qiagen, Carlsbad, CA, USA).

### 2.3. Flow Cytometry

A 50 μL volume of EhV-86 treated with 0.3% (final concentration) Termin-8 and 0.2% (final concentration) Finio, and non-treated EhV-86, were sampled and fixed with glutaraldehyde (0.5% final concentration), as described in Balestreri et al., 2024 [3]. All samples were subsequently diluted 1:100 with 1 mL final volume, stained with SYBR gold (1/100,000 final dilution), and analyzed on an Accuri C6 flow cytometer (BD Biosciences, San Diego, CA, USA) using an FL1-H threshold of 700 and fast fluidic rate (66 μL min^−1^).

### 2.4. Statistical Analysis

All V-qPCR results were analyzed via ANOVA with independent two-sample *t*-tests, as applicable in Excel 16.0 (Microsoft Corporation, 2018), to determine the significance of differences among treatments. Statistically significant differences between treatments were designated at a significance level of *p* ≤ 0.05.

## 3. Results

### Viability of EhV After Exposure to Temin-8 and Finio

**Figure 1:**
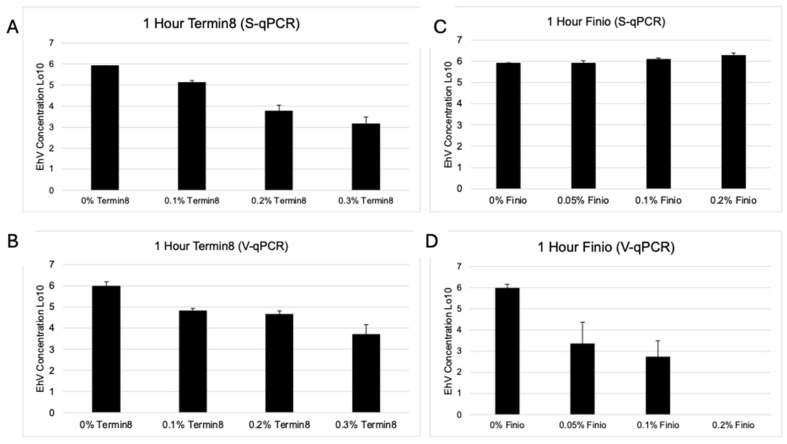
Average log_10_ EhV after 1 h exposure to 0.1%, 0.2% or 0.3% Termin8 (**A**,**B**) or 0.05%, 0.1% or 0.2% Finio (**C**,**D**) for S-qPCR (**A**,**C**) or V-qPCR (**B**,**D**). Bars = standard deviation.

After 1 h of treatment with Termin-8, there was a reduction in viral DNA (S-qPCR) equivalent to that observed in the V-qPCR assay, with the greatest reduction in viral DNA occurring at the highest concentration (0.3%, Figure 1A,B). While Finio showed negligible viral DNA reduction after an hour of treatment regardless of dose, as observed by S-qPCR, when Finio was added at the highest concentration (0.2%), there was complete inactivation (>5 log reduction units) of viable virus (Figure 1C,D).

**Figure 2:**
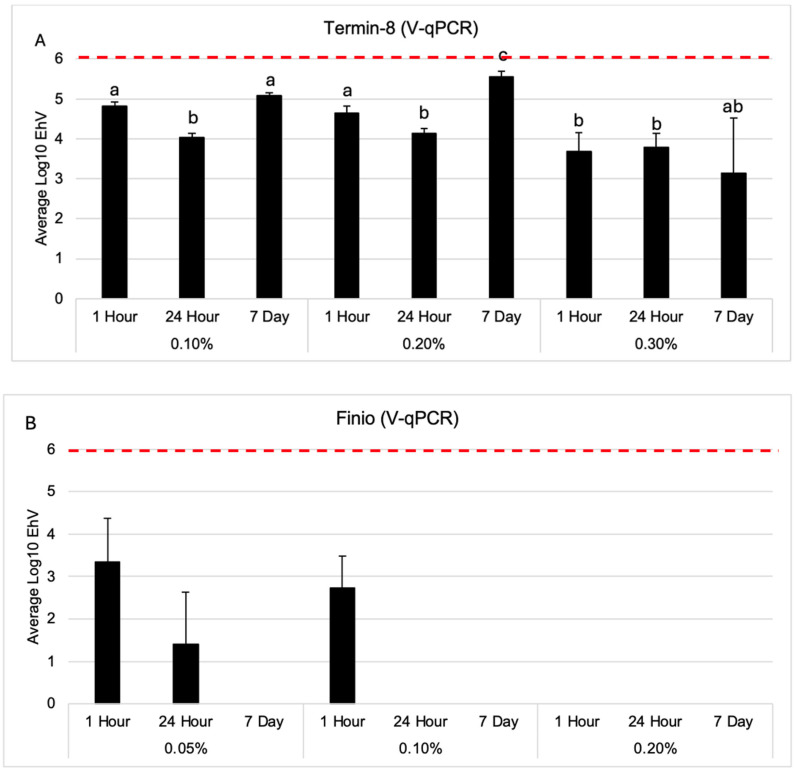
Average viral log_10_ EhV concentrations of Termin-8 (**A**) and Finio (**B**) at all time points. Red dotted line indicates the starting EhV log_10_ concentration. Bars = standard deviation. Differing letters denote statistical significance at *p* ≤ 0.05.

For all concentrations (0.1%, 0.2% and 0.3%) tested, Termin-8’s antiviral activity increased over a 24 h time exposure (Figure 2A). At 24 h, all concentrations (0.1%, 0.2%, and 0.3%) of Termin-8 resulted in significantly lower (*p* = 0.04, 0.05, and 0.05, respectively) concentrations of EhV compared with the virus concentrations from 0.1% Termin-8 at 1 h of exposure. After 7 days incubation at a concentration of 0.1% Termin-8, the amount of viable virus detected via V-qPCR was significantly greater than the 0.3% treatment at 1 h, and at all concentrations (0.1%, 0.2%, and 0.3%) after 24 h (*p* = 0.02, 0.01, 0.02, and 0.02, respectively). Furthermore, after 7 days of incubation, the amount of viable viral DNA exposed to 0.2% Termin-8 was significantly greater than the DNA at all concentrations at 1 h (*p* = 0.05, 0.04, 0.03), all concentrations at 24 h (*p* = 0.03, 0.03, and 0.03), and 0.3% Termin-8 at 7 days of incubation (*p* = 0.04). In summary, only 0.3% Termin-8 showed consistent antiviral activity over all the time exposures tested.

Over time, all tested concentrations (0.05%, 0.1%, and 0.2%) of Finio resulted in complete inactivation after 7 days of incubation, with a reduction of 5.90 log_10_ units (Figure 2B). Both the highest dose concentrations (0.3% and 0.2%) of Termin-8 and Finio, respectively, resulted in the greatest log reduction of 2 and 4.5 log_10_ units, respectively, at the earliest (1 h) time point (Figure 1 and Figure 2). At 24 h, all concentrations (0.1, 0.2, and 0.3%) of Termin-8 had a 1.97, 1.86, and 2.21 log_10_ unit reduction, respectively, but after 7 days of incubation, the lower dosages (0.1% and 0.2%) resulted in a less than 1 log_10_ unit reduction (Figure 2A).

**Figure 3:**
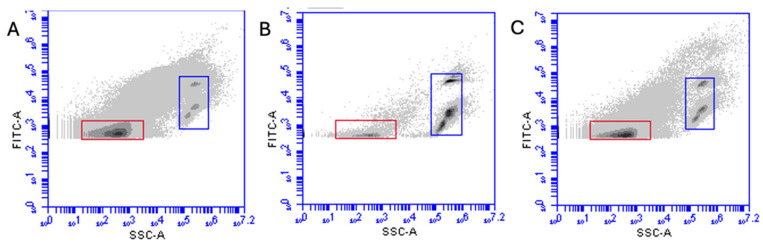
Flow cytometry plots of non-treated (**A**) and highest concentration of Finio (**B**) and Termin-8 (**C**), with red rectangles representing EhV population while blue rectangles represent control staining beads.

All these results were confirmed by flow cytometry analysis (Figure 3A–C). We found that 0.2% Finio reduced the amount of virus particles detected via flow cytometry (Figure 3B, red rectangle, 8.44 × 10^5^ EhV/mL), while the amount of EhV particles from the 0.3% Termin-8 treatment after 1 h (Figure 3C, red rectangle, 3.28 × 10^6^ EhV/mL) was similar to the amount of virus in non-treated samples (Figure 3A, red rectangle, 7.45 × 10^7^ EhV/mL).

## 4. Discussion and Conclusions

Previous research has shown that megaviruses, such as ASFV and the algal surrogate EhV, are very stable in the environment, particularly at high temperatures [3,5]. Additionally, these viruses have been shown to be relatively stable and can survive for up to 21 days in various feed ingredient matrices [1,2,4,5,8,9,10]. Although certain feed ingredients can undergo extremely high temperatures during various processing stages, a change in the amount of time and the temperature cannot fully inactivate the viral load [3,5]. Therefore, the U.S. feed and swine industry is interested in developing feed supply chain biosecurity protocols that include effective chemical mitigation strategies for swine viruses, including ASFV. The results from previous research have shown that viral inactivation by chemical mitigation may leave the host animal better prepared to deal with infection from that particular virus, yet the mechanism remains largely unknown [14,15]. In this study, we used the viability qPCR method to quantify the amount of intact or “viable” viruses left in a sample after exposure to chemical mitigants. Any degraded, damaged, or compromised viral particles were bound by photoreactive dye and unable to amplify during PCR amplification, thereby determining how many viral particles were left intact after chemical mitigation [16,17]. These viability results provide us with an idea of the potential for the virus to infect a host after chemical mitigation.

Currently, Termin-8 and Finio are commercially used to control microbial contamination in feed. In this study, we tested the antiviral activity of both compounds to show that they can be effective chemical mitigants of ASFV, which provides the swine industry with a valuable feed biosecurity strategy. Overall, Finio and, to a lesser extent, Termin-8 have the potential to be effective chemical mitigants against megaviruses such as ASFV, with the greatest log reduction of 2 (0.3% Termin-8) and 4.5 (0.2% Finio) log_10_ units observed after 1 h exposure to EhV. Additionally, the mechanism of chemical mitigation by both Temin-8 and Finio occurs through the degradation of the virus particle itself. Although the results from this experiment appeared to indicate that the efficacy of Termin-8 is lower than that of Finio, this is likely not the case because Termin-8 appears to cross-link and fix viral particles, making them viable but non-infectious [18]. However, future bioassay-based experiments are needed to confirm this mechanism. Additionally, further research in feedstuffs and ASFV to understand the full scope of how these compounds work in real-world scenarios is warranted.

## Data Availability

Data are contained within the article.
